# Financial burden impact quality of life among lymphatic Filariasis patients

**DOI:** 10.1186/s12889-021-10170-8

**Published:** 2021-01-21

**Authors:** Samuel Opoku Asiedu, Alexander Kwarteng, Emmanuel Kobla Atsu Amewu, Priscilla Kini, Bill Clinton Aglomasa, John Boulard Forkuor

**Affiliations:** 1 Kumasi Centre for Collaborative Research in Tropical Medicine, Kwame Nkrumah University of Science and Technology, Kumasi, Ghana; 2 Department of Biochemistry and Biotechnology, Kwame Nkrumah University of Science and Technology, Kumasi, Ghana; 3 Department of Sociology and Social Work, Kwame Nkrumah University of Science and Technology, Kumasi, Ghana

**Keywords:** Lymphatic Filariasis, Quality of life, Ghana

## Abstract

**Background:**

Human lymphatic filarial pathology is the leading cause of disability and poverty among people living with the infection. The second goal of the Global Programme to Eliminate Lymphatic Filariasis (GPELF) is to manage the disease’s morbidity to improve patients’ quality of life. Consequently, the current study assessed the overall quality of life of lymphatic filariasis (LF) pathology patients in some selected endemic communities in rural Ghana.

**Method:**

In the present study, the Lymphatic Filariasis Quality of Life Questionnaire (LFSQQ) was used to evaluate the effect of lymphatic filariasis on the quality of life of people, with the disease in nine (9) communities in the Ahanta West District of the Western Region of Ghana where mass drug administration is being implemented for the past twenty years. Pearson’s correlation, linear regression, and one-way analysis of variance (ANOVA) analyses were used to assess the associations between the LFSQQ instrument domains.

**Results:**

Of the 155 study participants recruited, 115 (74.19%) were females, and 40 (25.81%) males. A greater proportion of the study participants (40, 25.8%) were presented with stage two (2) lymphoedema, while only two patients had stage seven (7) lymphoedema. The average of the overall quality of life scores of study participants was 68.24. There was a negative Pearson correlation (*r =* − 0.504, *p-*value < 0.001) between the stage of lymphoedema (severity of the disease) and the quality of life of the LF patients. In addition, a clear pattern of positive correlation (*r =* 0.71, *p-*value < 0.001) was observed between the disease burden and pain/discomfort domains of the study participants. Whereas the highest domain-specific score (85.03) was observed in the domain of self-care, we noted that the environmental domain, which consists of the financial status, was the lowest (45.94) among the study participants.

**Conclusion:**

Our findings support previous works on the reduced quality of life among lymphatic filariasis patients with pathology. In this study, our results reveal a depressing financial condition among people presenting with late stages of LF pathologies, which eventually reduces their well-being.

## Background

Lymphatic filariasis (LF) is an infection that directly impairs the lymphatics and renders long-lasting disability to its victims [1]. LF is a mosquito-borne disease in which mosquitoes transmit the causative organisms (*Wuchereria bancrofti*, *Brugia timori* and *B. malayi* filarial worms) [2] to uninfected persons. The disease is endemic in 83 countries, with most of the cases reported in India, one third in Africa, and the remaining cases in the Pacific, the Americas, South-East Asia, and the Eastern Mediterranean regions [3]. In Ghana, bancroftian filariasis has been noted to be distributed in the northern guinea savannah and the southern coastal regions with a varied microfilaraemia prevalence of 0–20% as surveyed in 1994 [4]. LF has a varied form of manifestations, such as hydrocele, lymphoedema, and elephantiasis [5]. For instance, in highly endemic communities such as Kassena Nankana District (Upper East Region of Ghana), the prevalence of hydrocoele and elephantiasis of the leg has been recorded to be about 31 and 3.8%, respectively [6]. In addition, women are ten times more likely than men to have lymphoedema of the leg [7]. This trend is still consistent in other neglected tropical diseases such as trachoma where female caregivers most often contact infected children than their male counterparts and thus are at higher risks of infecting themselves [8]. Individuals suffering from lymphatic filariasis experience repeated filarial attacks known as adenolymphangitis (ADL), which hinders them from actively participating in social and economic activities [9–11]. Krishna et al. 2005, identified ADL as the primary cause of disabilities among LF patients [12].

In line with the strategies of the Global Programme for Elimination of Lymphatic Filariasis (GPELF) to interrupt LF transmission in endemic areas, more than 890 million individuals have participated in the mass drug administration (MDA) programmes in 37 countries as of 2017 [5]. In Ghana, MDA was started in 2001 and had reached national coverage in 2006. After 14 years of the annual treatment of MDA, 69 districts had achieved low transmission of the disease; thus, MDA was stopped for these districts, 29 districts still have persistent transmissions. The persistent transmission of the disease in such districts is not clear; however, in Tanzania, a similar trend was observed. This was attributed to poor coverage of MDA and non-compliance to the medication by a section of the population.

Nonetheless, the second component of the LF elimination programme of forestalling and managing acute and chronic disability among those already affected by the disease has not achieved appreciable results. The idea of this strategy is to assist the 40 million people already affected by the disease and largely neglected, to have a better quality of life, and to be capable of engaging fully in both economic and social activities [5]. Quality of Life is defined as an individual’s perception of one’s position in life in the context of value systems and culture, and in relation to one’s goals, standards, expectations, and concerns [13]. Thus, in this perspective, WHO defines health as being “not only the absence of disease and infirmity but also the complete state of physical, mental, and social well-being” [14].

To ensure a better health-related quality of life of LF patients, the Morbidity Management and Disability Prevention (MMDP) of the World Health Organization (WHO) enrolled a basic package of care. This package of care must be accessible to LF patients, i.e., surgery for hydrocele, treatment for episodes of adenolymphangitis, management of lymphoedema to hinder episodes of adenolymphangitis, and progression of disease [15]. To this end, GPELF initiated the Community Home-Based Care (CHBC) concept of reaching out to individuals with varying degrees of morbidities to alleviate pain and prevent disease deterioration [16]. Thus, this study aimed to assess the quality of life of lymphatic filariasis patients in some selected LF hotspot communities in Ghana.

## Methods

### Study area

The study was carried out in the Ahanta West District of Western Ghana in nine (9) communities, i.e., Akatakyi, Princess Town, Cape Three Points, Asemkow, Dixcove, Ampatano, Butre, Achowa and Busua. The District is dotted with lush green hills and fertile soil with relative humidity as high as 85% in the rainy season and a slight decline in humidity during the dry season. A bulk of the labour force in these villages is into activities such as fishing and farming. The study communities are lymphatic filariasis hotspots, with *W. bancrofti* being the main causative organism and a microfilariae prevalence of about 20% [17].

### Study design

A cross-sectional study was conducted between March 2019 and August 2019. Individuals clinically diagnosed with lymphatic filarial pathology (lymphoedema and/or hydrocele) were recruited for the study. In addition, the study participants were 18 years and above and willingly consented to the study. However, individuals presenting with any form of swelling or edema other than filarial-related were excluded from the study. Experienced research scientists performed the leg staging of the study participants. The WHO seven-stage system for grading lymphoedema was used as the standard for grading the patients’ legs as previously described in [9]. The Committee of Human Research and Publications and Ethics, School of Medical Sciences and Dentistry, Kwame Nkrumah University of Science and Technology provided ethical clearance for this study.

### Study instrument

To assess the LF study participants’ quality of life, the Lymphatic Filariasis Quality of Life Questionnaires (LFSQQ) was used as described by Thomas, et al. (2014) [18]. The LFSQQ was administered to the study participants in their local dialects (Fante and Nzema). The instrument measured the health-related quality of LF patients through a seven-domain system: mobility, self-care, daily/usual activities, disease burden, pain/discomfort, psychological health, and social participation. However, for this study, energy/fatigue, environment (which entails financial support from people in the community and safety in the neighborhood), and social relationship domains were included due to the extended family system in most Ghanaian communities. Each item was scored on a 5-point scale (no problem, mild, moderate, severe, most severe). The total score was calculated based on the number of questions answered, and the raw scores. Scores range from 0 to 100, with a higher score indicating a better quality of life described by Aggithaya, M.G et al.*, 2013*. In addition, the score range was further categorized arbitrarily; (0–50) as low quality of life, (51–69) as the moderate quality of life, and (70–100) as high quality of life.

### Statistical analyses

Data were analyzed using the Epi-Info. To do this, the overall quality of life (QoL) response was calculated using the formula as described by Aggithaya, M. G et al.*, 2013*.

$$ \mathrm{Overall}\ \mathrm{QoL}=\frac{\mathrm{Total}\ \mathrm{Score}\mathrm{s}}{\mathrm{Higest}\ \mathrm{Score}\ (5)\times \mathrm{Number}\ \mathrm{of}\ \mathrm{Questions}\ \mathrm{Anwered}}\times 100\% $$

The Domain score calculated using the formula,

$$ \mathrm{Domain}\ \mathrm{score}=\frac{Total\ score\  on\  domain}{Highest\ Score\ (5)\times Number\ of\ Questions\ Answered}\times 100\% $$

To determine the relationship between the LFSQQ domains of the study instruments, the Pearson Correlation analysis was performed. After that, linear regression analysis was done to examine the association between disease burden and the pain/discomfort domains. A one-way analysis of variance (ANOVA) was used to determine whether there was any statistical difference in the disease burden domain, psychological domain, and environment domain. The proportion of the domain scores of study participants are given in percentages and mean. Any result with a *p*-value less than 0.05 was considered statistically significant.

## Results

### Demography of study participants

Together, 155 study participants were recruited. Three-quarters (115, 74.19%) of the study participants were females and the remaining (40, 25.81%) males. The mean age of the study participants was 52.84 (SD = 15.62) with the age range of (18–86). The majority of the study subjects (97, 62.58%) were involved in agrarian and/or fishing activities while occupations such as service workers, sales workers, and professional related workers (5, 3.23%; 12, 7.74%; 2, 1.29%), respectively, was less common among the LF cohorts. Thirty-nine (39) of LF subjects representing 25.16%, were unemployed, as Table 1. A greater number of LF patients (84, 54.19%) in this study had stage 2 lymphoedema (swelling not reversible overnight). The patients with stage 1 and stage 3 were 17 (10.98%) and 28 (18.06%), respectively. Two (2) of the study participants with stage 7 were incapacitated and could not care for themselves, whereas the remaining were with other stages, as shown in Table 2.

**Table 1:** Demography of Study Participants

SEX	n (%) ^**a**^
Female	115 (74.19)
Male	40 (25.81)
**AGE**	
(mean ± SD)	52.84 ± 15.62 **95%CI** (50.34–55.33)
**Occupation**	
Agricultural/Fishman/Fishmonger/Farmer	97 (62.58)
Service workers	5 (3.23)
Sales workers	12 (7.74)
Professionals/Pensioner /teacher	2 (1.29)
Unemployed	39 (25.16)
**Community**	
Achowa	4 (2.58)
Akatakyi	37 (23.87)
Ampatano	21 (13.55)
Asemkow	24 (15.48)
Busua	13 (8.39)
Butre	14 (9.03)
Cape 3 points	10 (6.45)
Dixcove	16 (10.32)
Princess Town	16 (10.32)

This table shows the various sections of the demography of the study participants with **CI** as a Confidence interval, ^**a**^ Percentage (%) is the number (n) divided by 155 (total N)

**Table 2:** Clinical profile of the patients

Stages of Lymphoedema	n(%)^a^	
1	Swelling reversible overnight	17(10.98)
2	Swelling not reversible overnight	84(54.19)
3	Shallow skin folds	28(18.06)
4	Skin knobs	6(3.87)
5	Deep skin folds	10(6.45)
6	Presence of “mossy lesions”	8(5.16)
7	Unable to care for self	2(1.29)

This table depicts the staging of the lymphoedema of the study participants, ^**a**^ Percentage (%) is the number (n) divided by 155 (total N)

### Quality of life score of LFSQQ domains among study participants

**Fig. 1:**
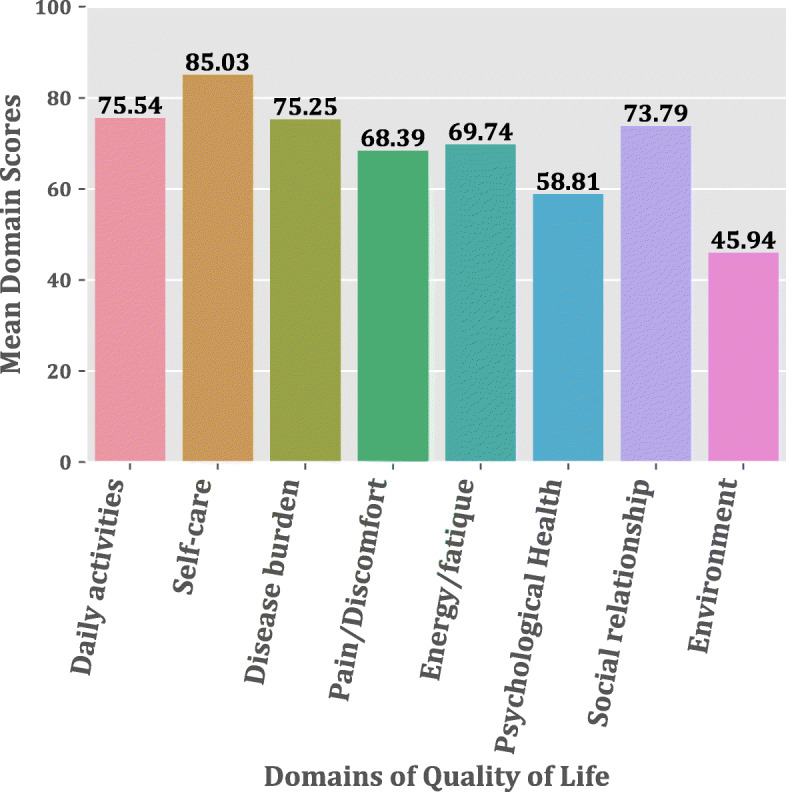
Bar Chart depicting the mean quality life scores of LF patients for each domain

The average overall quality of life score among the LF cohort was 68.24 (SD = 9.76). Among the domains used in the study, LF pathology patients recorded the Environment domain as the lowest quality of life score (45.94), while the self-care domain being the highest quality of life score (85.03). The study instrument’s Daily activities domain and Disease burden domain had a slight difference in their scores of 75.54 and 75.25, respectively. A similar trend was also observed between the Mobility domain and Pain/Discomfort domain (Fig. 1).

In addition, over 80% of the study participants responded to not having “no problem with their ability to take care of themselves” (self-care). In contrast, over 50% of LF patients complained of severe to most severe effects of the disease on their work and loss of strength or fatigue. In the Mobility domain, the study participants had severe problems when they sat and got out of a chair or standing for a long time. With regard to their daily activities, the majority of LF patients (74%) did not have any challenge with cooking or cleaning the floors, but they had difficulties in carrying out activities such as fishing or farming. Moreover, from the Social relationship domain’s responses, an average of 58% do not have any issue interacting with people, identifying a potential spouse, or having the disease affecting their relationships with their family members or neighbors. The Environment domain response showed that 67% of respondents indicated little or no financial assistance when asked “how often do they get financial assistance from their relations due to their conditions”?

### Quality of life score of LFSQQ domains in the community level

**Fig. 2:**
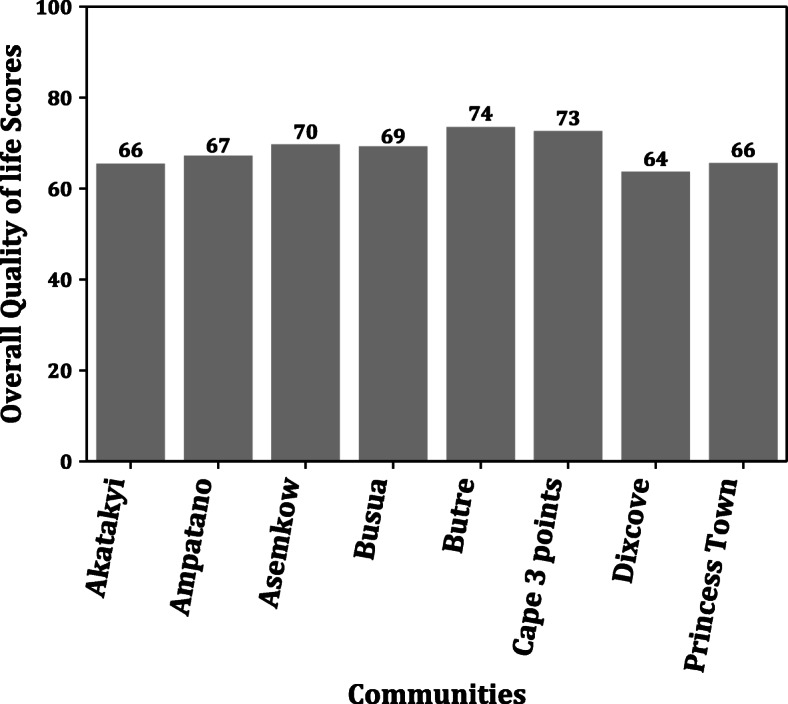
The Overall Quality Life Scores in the Communities

**Fig. 3:**
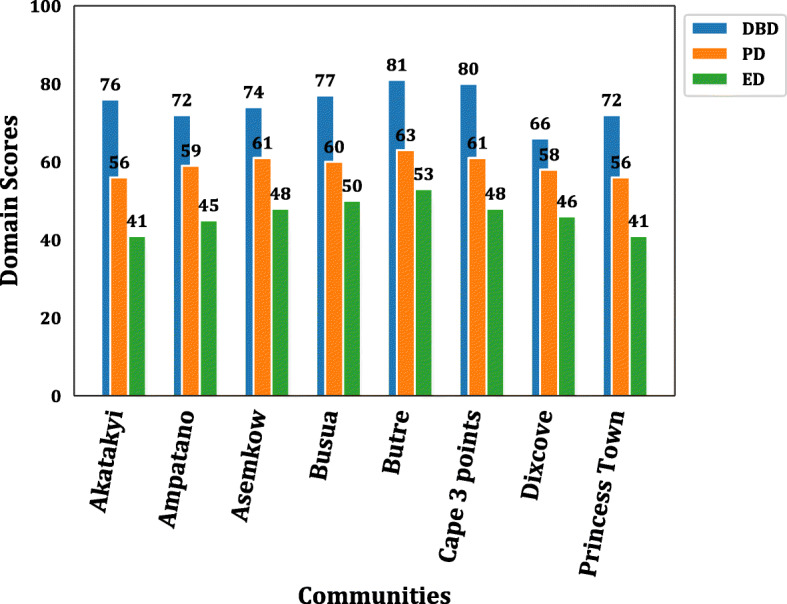
The Distribution of Domains Scores in the Community. This graph shows the domains scores of DBD, PD, and ED in the community of study. The bar value represents the domain scores for DBD, PD, and ED for each of the study community. The scores range from 0 to 100 with a higher score indicating a better quality of life. *DBD: Disease Burden Disease, * PD: Psychological Domain, *ED: Environment Domain

The nine (9) communities where the study was conducted had a varied number of LF patients, as shown in Table 1. To determine the overall quality score in the various LF endemic communities, a bar graph of the overall quality score among the communities was done, as demonstrated in Fig. 2. The graph showed marginal changes in the overall quality of life scores of LF patients residing in the communities. However, the Achowa community, which had only four (4) LF patients, was excluded from the analysis to give more representative data. Moreover, considering that the Disease Burden Domain (DBD), Psychological Domain (PD), and Environment Domain (ED) were the lowest domains in the LFSQQ, a community-level distribution of these domains scores, i.e., DBD, PD, and ED were analyzed. As shown in (Fig. 3), there was generally a higher score in the DBD within the range 76–80 except for Dixcove, where the DBD score of 66. A similar trend is also observed in ED and PD with the ranges of 41–53 and 56–63, respectively. However, to determine whether there was any statistical difference among the DBD, PD, and ED in the communities, a one-way analysis of variance (ANOVA) was conducted, which showed statistical difference in these domains in the community (F (2, 24) = 73.06, *p*-value < 0.001) as shown in Table 3. In addition, the overall quality score in the female and male was 68.42 and 67.61, respectively.

**Table 3:** One-Way Analysis of Variance (ANOVA) of Domain Score

	df	Sum_sq	Mean_sq	F	***P-***value
**C (Domain score)**	2	3870.888889	1935.444	73.06117	< 0.001
**Residuals**	24	635.7777778	26.49074		

### Correlation of LF responses on LFSSQ domains

**Fig. 4:**
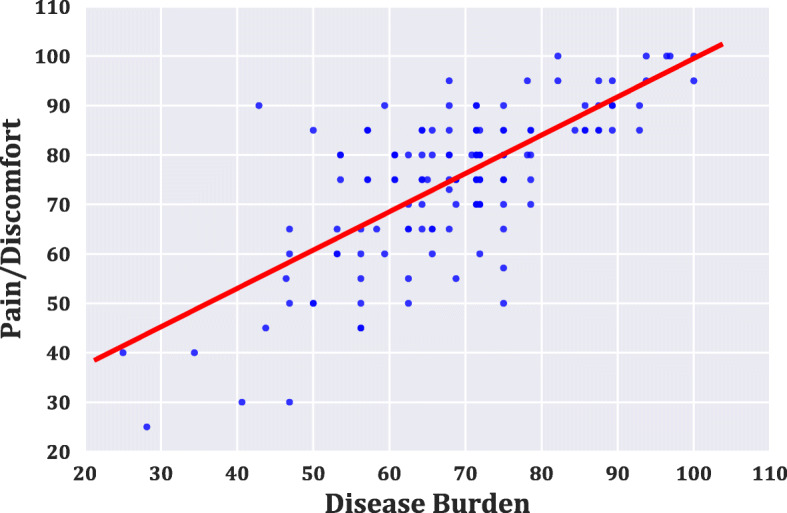
The Correlation result between the Disease Burden and Pain/Discomfort Domains. The graph shows a positive Pearson’s correlation (*r =* 0.71, *p-*value < 0.001) between the Pain/Discomfort domain and Disease burden domain

**Fig. 5:**
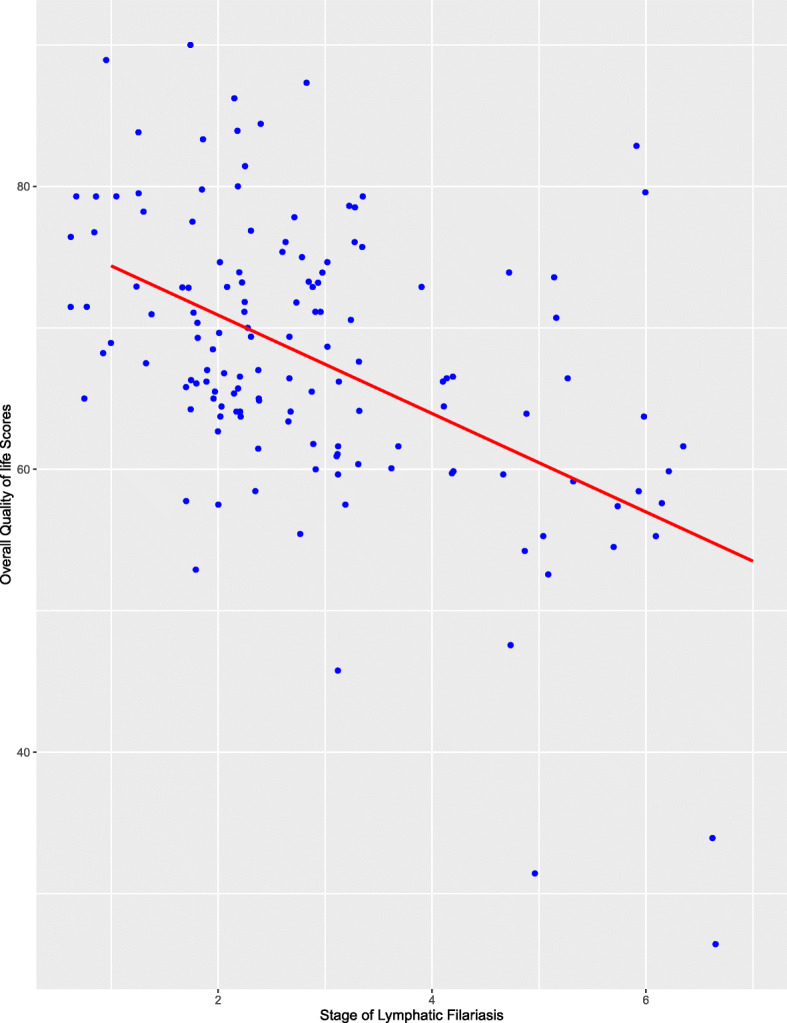
The correlation result of the severity of disease and overall Quality of Life Scores. The graph depicts a negative linear regression between the overall quality of life score and LF stage (severity of disease) with a Pearson’s correlation coefficient (r) of − 0.504

To determine the relationship among the various LFSSQ domains, we performed Pearson’s correlation, as illustrated in Table 4. The domains of pain/discomfort and disease burden were positively correlated with a Pearson’s correlation coefficient of *r =* 0.71 and a *p*-value < 0.001 as depicted in Fig. 4. A number of the domains were moderately correlated with Pearson’s correlation coefficient (r), ranging from 0.60–0.66. The weakest correlation coefficient was observed between the domains of environment and pain/discomfort. Moreover, the linear regression model for the stage of LF (severity of the disease) and the overall quality of life score showed a negative regression with a Pearson’s correlation coefficient (*r =* − 0.504) and *p*-value < 0.001 (Fig. 5). In addition, to predict how the stage of LF (severity of the disease) and the overall QoL of participants influence the sex outcome. We performed a logistic regression analysis using the sex of the study participants as the dependent variable (y) and the stage of LF and overall QoL as the two independent variables (x). The *p*-values of the LF stage and the overall QoL as the two independent variables in the model were 0.015 and 0.419, respectively, as shown in Table 5. Thus, the association between the dependent variable (Sex) and the independent variable (stage of LF) is significant.

**Table 4:** Pearson’s Correlation for the Domains of Quality of Life Scores LF Patients

Variables	1	2	3	4	5	6	7	8	9
**1**. Mobility	–								
**2**. Daily Activities	0.62^b^	–							
**3**. Self-care	0.65^b^	0.60^b^	–						
**4**. Disease/ Burden	0.65^b^	0.56^c^	0.58^c^	–					
**5**. Pain/Discomfort	0.66^b^	0.50^c^	0.56^c^	0.71^a^	–				
**6**. Work/Fatigue	0.63^b^	0.62^b^	0.64^b^	0.63^b^	0.58^c^	–			
**7**. Psychological Health	0.43	0.41	0.50^c^	0.40	0.45	0.48	–		
**8**. Social Relationships	0.49	0.53^c^	0.54^c^	0.53^c^	0.46	0.56^c^	0.53^c^	–	
**9**. Environment	0.24	0.32	0.23	0.16	0.09	0.23	0.47	0.51^c^	–

^a^ Strong Correlation, ^b^ Moderate Correlation and ^c^ Weak Correlation

**Table 5:** Logistic regression model for dependent variable Sex

Variable	coef	Std. Error	z	*P* > |z|	[0.025	0.975]
const	3.2185	1.686	1.909	0.056	−0.087	6.524
QoL	−0.0168	0.021	−0.808	0.419	−0.058	0.024
Stage of LF	−0.3540	0.145	−2.436	0.015	− 0.639	−0.069

## Discussion

This is the first study to determine the general quality of life of lymphatic filariasis patients living in Ghana, even though a similar study has been conducted in India [1]. From our results, the overall mean QoL score for the LF subjects was 68.24, which is almost the same as the study conducted in India [19] at the baseline. Nevertheless, the quality of life of LF participants in this study was slightly lower than a previous study conducted in the urban area of Tiruchirappalli in India, where an overall mean QoL score of 69.81 [1] using the same LFSQQ instrument. Even though a score of 100 is the highest quality of life, our study participants’ overall QoL of 68.24 presents a good quality of life. Notwithstanding, a higher QoL could be achieved if LF patients are taken through regular skin wash activities, exercises, and other requisite hygienic practices. A six (6) months camp set up for LF patients in three endemic communities in India’s Kerala province showed a significant upsurge in the QoL of the patients from 68.23 to 74.57 [19].

Moreover, more than half of our LF participants responded that they had no financial assistance from any relation. This was not surprising since the environment domain was observed to have the least domain QoL score. There are apparent reasons for such observations. First of all, the observation can be due to the dilapidating consequences of lymphatic filariasis on the patients, which rendered most of our study participants jobless or had reduced working days, making them wholly dependent on relatives and friends. This finding confirms other results from this study, where 29% of the respondents indicated that their condition had a negative effect on their ability to engage fully in economic activities. In addition, the stigma associated with the disease prevented some of the study participants from embarking on their jobs, particularly those who were engaged in fish mongering and petty trading. The situation is further complicated as the LF usually affects poorer communities [12, 20] where most LF patients’ mainstay financial support (relatives and friends) is most often limited. This reason is corroborated by responses given by LF infected individuals in a different study conducted in Togo [21]. Our results show that 78% of the respondents felt insecure in their routine life. Although the reason for this insecurity was not immediately clear from our study, this may be due to the accustomed dejection and infamy most individuals who suffer neglected tropical disease face in their respective communities [12].

A community-level analysis of the overall quality of life also revealed that the QoL score did not depend on which community the LF patients resided in as it tends not to show any vast differences across the communities of study. This may be due to similar economic statuses of the patients across the communities, which tend to be an upshot of LF disease’s impact, making them depend on peasant farming and/or fishing activities for their livelihood. An observed general pattern of the specific domains, i.e., Psychological, Disease burden, and Environment domains in LFSQQ across the communities, also consolidated the impression that the LF patients in this study experienced almost the same impact infection of the community origin. Therefore, any intervention developed to alleviate the suffering and/or to increase the quality of life of LF patients can be generalized for all and possibly replicated in other similar settings.

The stage of the lymphoedema defined the severity of the disease. The worth of wellness of the study participants presenting the late stages of the infection was abysmal as the indicators observed declined in their overall mean QoL index. The negative Pearson’s correlation (*r =* − 0.504, *p*-value = 0.001) between the severity of the disease and the quality of life of the study participant was divergent with the positive correlation (*r =* 0.74, *p-*value < 0.001) of the severity of disease and quality of life score in a different study [1]. Nevertheless, an erstwhile study using modified Dermatology Life Quality Index (mDLQI) also revealed that the severity of the filarial lymphoedema had a considerable negative repercussion on the QoL of the individuals [22]. A possible explanation for the negative effect of the stage of the lymphoedema on the respondents’ quality of life in our study may lie in the fact that as the swelling of their affected limbs worsened, the less productive they become and so a toiling effect on the well-being of LF patients. Besides, the stage of LF is in a close association with the Sex of the study participants using the logistic regression model (*p-*value = 0.015).

For further analysis of how the disease impacts the LF participants’ physical abilities, we used the mobility, daily activities, pain/discomfort, and self-care domains in assessing this as used elsewhere [12]. The physical ability of LF participants has been widely reported to be hampered as compared to healthy individuals without the condition [5, 12, 15, 16], and this was expected in this study. On the other hand, other studies [1, 19] have shown a higher level of self-care among LF patients, which corroborates with our current study, where the self-care domain had the highest average QoL score of 85.03. This finding reflects the composition of our LF patients in the study, where 36% of them live with stage two (2) lymphoedema and could still take care of themselves.

Mobility of LF patients is also considered a key factor in assessing the patients’ physical capabilities as the various manifestations of the disease (ADL, hydrocele, lymphoedema, and elephantiasis). This situation has a cascading effect on the employment and income status of persons living with the condition [23, 24]. However, our study participants averagely had a mobility domain score of 68.86, which is comparatively higher to previous studies of 43.1 [20], 54.92 (baseline score) [19], and 52.32 [1]. As an earlier study have also detailed the mobility of LF patients by using a 10 m walking test (10mWT) and a timed ‘up and go’ (TUG) test revealed LF patients were slower than controls (10WT: cases = 0.828 m/s, controls = 1.104 m/s, TUG: cases =14.7 s, controls =11.2 s).

This study was conducted in rural areas along the western coast of Ghana, with a higher proportion of female participants. While this observation is not uncommon in LF endemic communities, care must be taken when the study’s findings are generalized to other LF endemic communities, particularly outside Ghana. A previous study has reported women or girls to be more prone to neglected tropical diseases (NTD), which may be due to higher poverty levels, higher illiteracy rates, and lower social status [8].

## Conclusion

This study, carried in the LF hotspot communities in the Western Region of Ghana, shows a challenge in the quality of life of lymphatic filariasis patients due to the condition. Our findings also reveal financial and income difficulties among people suffering from LF, which eventually reduces their socio-economic well-being. We provide compelling evidence that there is a need for relevant governmental and non-governmental stakeholders, including the Ministry for Gender and Social Protection of Ghana, to integrate some social intervention programmes for people living with LF conditions in endemic regions to improve their well-being. We believe the study will inform policy about the management of LF in Ghana.

## Data Availability

The raw data may be made available upon reasonable request from the corresponding authors.

## Abbreviations

GPELF: Global Programme to Eliminate lymphatic filariasis
LF: Lymphatic Filariasis
LFSQQ: Lymphatic Filariasis Quality of life Questionnaire
ANOVA: Analysis of Variance
ADL: Adenolymphangitis
MDA: Mass Drug Administration
MMDP: Morbidity Management and Disability Prevention
CHBC: Community Home-Base Care
QoL: Quality of Life
DBD: Disease Burden Domain
PD: Psychological Domain
ED: Environment Domain
WT: Walking Test
TUG: Timed ‘Up and Go’
